# Differential microRNA Analysis of Glandular Trichomes and Young Leaves in *Xanthium strumarium* L. Reveals Their Putative Roles in Regulating Terpenoid Biosynthesis

**DOI:** 10.1371/journal.pone.0139002

**Published:** 2015-09-25

**Authors:** Rongyan Fan, Yuanjun Li, Changfu Li, Yansheng Zhang

**Affiliations:** 1 CAS Key Laboratory of Plant Germplasm Enhancement and Specialty Agriculture, Wuhan Botanical Garden, Chinese Academy of Sciences, Wuhan, China, 430074; 2 Graduate University of Chinese Academy of Sciences, Beijing, China, 100049; Nanjing Agricultural University, CHINA

## Abstract

The medicinal plant *Xanthium strumarium* L. (*X*. *strumarium*) is covered with glandular trichomes, which are the sites for synthesizing pharmacologically active terpenoids such as xanthatin. MicroRNAs (miRNAs) are a class of 21–24 nucleotide (nt) non-coding RNAs, most of which are identified as regulators of plant growth development. Identification of miRNAs involved in the biosynthesis of plant secondary metabolites remains limited. In this study, high-throughput Illumina sequencing, combined with target gene prediction, was performed to discover novel and conserved miRNAs with potential roles in regulating terpenoid biosynthesis in *X*. *strumarium* glandular trichomes. Two small RNA libraries from leaves and glandular trichomes of *X*. *strumarium* were established. In total, 1,185 conserved miRNAs and 37 novel miRNAs were identified, with 494 conserved miRNAs and 18 novel miRNAs being differentially expressed between the two tissue sources. Based on the *X*. *strumarium* transcriptome data that we recently constructed, 3,307 annotated mRNA transcripts were identified as putative targets of the differentially expressed miRNAs. KEGG (Kyoto Encyclopedia of Genes and Genomes) pathway analysis suggested that some of the differentially expressed miRNAs, including miR6435, miR5021 and miR1134, might be involved in terpenoid biosynthesis in the *X*. *strumarium* glandular trichomes. This study provides the first comprehensive analysis of miRNAs in *X*. *strumarium*, which forms the basis for further understanding of miRNA-based regulation on terpenoid biosynthesis.

## Introduction

MicroRNAs (miRNAs) are small non-coding, endogenous RNAs consisting of ~22 nt in average, and are generated from large stem-loop precursors transcribed from non-protein-coding genes, introns or coding regions of the host genome[1, 2]. They interact with mRNAs through perfect or non-perfect complementarity to degrade mRNAs or repress translation, thus negatively regulating gene expression post-transcriptionally [3–7]. Plant miRNAs have been reported to be involved in various biological processes, including plant growth development, signal transduction, and stress responses against biotic or abiotic factors [8–12]. They also target genes with functions in metabolite biosynthesis [13,14]. With the aid of a high-throughput sequencing technology, there are increasing miRNAs identified and characterized from a number of medicinal plant species, e.g. Panax ginseng [15], Opium poppy [16], Taxus [17], and Catharanthus roseus [18], and their roles in regulating the production of secondary metabolites of interests were also suggested by the bioinformatics analysis. In the model plant *Arabidopsis thaliana*, there was direct evidence that miRNAs regulated the biosynthesis of secondary metabolites by modulating their expression *in vivo*. For instance, the overexpression of miRNA393 in *Arabidopsis thaliana* altered the levels of glucosinolate and camalexin via perturbing the auxin signaling pathway [19]. Other studies showed that the flavonoid biosynthesis of *Arabidopsis thaliana* was regulated by the expression of miRNA156 while the modulation of miRNA163 expression level changed the profiles of secondary metabolites [20, 21].

*Xanthium strumarium* L. (*X*. *strumarium*), an annual growth herb, belongs to the compositae family [22]. The whole plant, especially its leaf, root and fruit, has been used in traditional medicine for the treatment of rhinitis, malaria, rheumatism, tuberculosis, cancer, and ulcers [23–26]. Previous studies indicated that plants of the Asteraceae family are characteristically rich in sesquiterpene lactones, an important class of terpenoids, and the *Xanthium*s species are rich in such medicinal ingredients [27–31]. The pharmacological properties of *X*. *strumarium* are largely attributed to the presence of xanthanolides (a class of sesquiterpene lactones), which have been reported to possess antifungal, antibacterial, and cytotoxic activities, and exhibit a growth inhibitory activity against insects [30, 32–37]. Despite their multiple bioactivities, especially their anti-tumor and anti-cancer activities [38, 39], the knowledge on how xanthanolides are biosynthesized and how the pathway is regulated remains largely unknown. Answering this scientific question is one of the long-term aims in our laboratory. Previously, we discovered that xanthanolides were highly biosynthesized and accumulated in the glandular trichomes of the *X*. *strumarium* tissues, especially on its leaves at early stage [40]. To identify genes encoding enzymes involved in trichome-dependent biosynthesis of xanthanolides in *X*. *strumarium*, the transcriptome dataset from two related tissue sources—glandular cells isolated from young leaves and intact young leaves was recently analyzed by our group. To study the regulatory mechanisms of xanthanolides biosynthesis, we focused our attentions on miRNA-based regulations as there are increasingly published literatures reporting their roles in plant secondary metabolic activities [15–19]. To date, reports on miRNAs in *X*. *strumarium* remain lacking. In this study, *X*. *strumarium* miRNAs were firstly identified using high-throughput sequencing technology and the differentially expressed miRNAs between the isolated glandular cells and intact young leaves were discovered. Combined with the analysis of the *X*. *strumarium* transcriptome, the targets of those differentially expressed miRNAs were predicted and their functions were annotated, which suggested that some of the differentially expressed miRNAs might play roles in regulating terpenoid biosynthesis in *X*. *strumarium* glandular cells.

## Materials and Methods

### Plant materials

Young leaves (the first and second leaves from the top) were randomly collected from different individual *X*. *strumarium* plants grown at the Wuhan Botanical Garden, Chinese Academy of Sciences, Wuhan, China (Aug. 10th, 2013). The age of the *X*. *strumarium* was three month-old. *X*. *strumarium* glandular trichomes were isolated from 20g of intact young leaves according to protocols described previously by Chen *et al*. with some modifications [40]. The young leaves were abraded in a cell disrupter (Bead-Beater, BIOSPEC, USA) using glass beads in an isolation buffer (25 mM MOPSO, pH 6.6, 200 mM sorbitol, 10 mM sucrose, 5 mM thiourea, 2 mM dithiothreitol, 5 mM MgCl_2_, 0.5 mM sodium phosphate, 0.6% (w/v) methylcellulose and 1% (w/v) polyvinylpyrrolidone). The disrupted extracts were filtered through a 425 μm nylon mesh, and the filtrate was then consecutively passed through 125, 80 and 42 μm nylon meshes with a resin buffer (25 mM MOPSO, pH 6.6, 200 mM sorbitol, 10 mM sucrose, 5 mM thiourea, 2 mM dithiothreitol, 5 mM MgCl_2_ and 0.5 mM sodium phosphate). The isolated glandular trichomes were retained on the 42 μm mesh. Each sample was flash frozen in liquid nitrogen and then stored at −80°C for RNA isolation.

### Small RNA library construction and high-throughput sequencing

Total RNA was extracted from fresh young leaves and the isolated glandular trichomes with Trizol reagent (Ambion). The quantity and quality of RNA samples were measured by Eppendorf BioPhotometer plus to ensure that the OD260/OD280 values were between 1.8 and 2.2. The RNA integrity was examined by agarose gel electrophoresis. Small RNA sequencing was performed using an Illumina Genome Analyzer at the Beijing Genomics Institute (BGI, Shenzhen, China). Small RNA fractions with the length range from18 to 30 nt were purified and then ligated to a 5' and 3' adaptor. After the reverse transcription followed by 11 cycles of polymerase chain reactions, approximately 20 μg of the amplified products were used for sequencing.

### Analysis of the sequenced data of the small RNAs

Small RNA reads with a length of 49 nt were produced by Illumina. Then data processing analysis was conducted as follows: (1) Removal of low-quality reads (more than four bases with sQ values below 10, and more than six bases with sQ values less than 13); (2) Removal of reads with 5′ adaptor contaminants; (3) Removal of reads without 3′ primer; (4) Removal of reads without an insert tag; (5) Removal of reads with poly A; (6) Removal of reads shorter than 18 nt; and (7) A summary of the length distribution of the clean reads. The remaining clean reads were mapped to *X*. *strumarium* transcriptome with less than two mismatches to analyze the expression and distribution of the small RNAs using SOAP software[41].To annotate the small RNAs, the sequences were aligned to the NCBI GenBank (http://www.ncbi.nlm.nih.gov/genbank/) and Rfam (http://rfam.sanger.ac.uk/) 10.1 databases by a BLAST search[42, 43]. The matched tags, including rRNA, scRNA, snoRNA, snRNA, and tRNA were eliminated. The remaining tags were used to detect conserved and novel miRNAs. The transcriptome databases of the *X*. *strumarium* small RNAs and mRNAs were deposited at the sequence read archive (SRA) of National Center for Biotechnology Information (NCBI) under the accession numbers of SRP056720 and SRP056511, respectively.

### Identification of the conserved miRNAs

There is no miRNA information for *X*. *strumarium* in miRBase. To identify the conserved miRNAs, the following strategy was used: first, considering the differences between species, clean data was aligned to mature miRNAs or miRNA precursors of all plants in miRBase 20.0 (http://www.mirbase.org)[44] allowing two mismatches using tag2miRNA software (developed by BGI); second, we chose the most abundant miRNA from each mature miRNA family to construct a temporary miRNA database; third, we aligned the clean data to the above temporary miRNA database and the expression of miRNA was generated by summing the count of all tags which were aligned to the temporary miRNA database within two mismatches. The small RNAs that were unaligned to any databases were defined as unannotated sequences.

### Prediction of the novel miRNAs

The unannotated sequences ranging from 18 to 25 nt were used to identify novel miRNAs by Mireap software based on the following main criteria described by chen et al. [45]: (1) The tags which could be used to predict novel miRNAs came from the set of unannotated tags which were matched to the transcriptome of *X*. *strumarium*; (2) Those tags whose sequences and structures satisfied the two criteria: hairpin precursors could fold into secondary structures and the sequences were present in one arm of the hairpin precursors, will be considered as candidate novel miRNAs; (3) Hairpin precursors lack large internal loops or bulges; (4) The secondary structures of the hairpins are steady, with the free energy of hybridization lower than or equal to -18 kcal/mol; (5) The number of mature miRNAs with predicted hairpin precursors must be at least five in the alignment results.

### Target gene prediction of the conserved and novel miRNAs

To obtain putative target genes, we matched the identified miRNAs to the *X*. *strumarium* transcriptome according to the rules published by Allen *et al*.[3] and Schwab *et al*.[7]. The criteria were (1) the number of mismatches between small RNAs and their targets should be less than four (G–U pairs count as half mismatch); (2) no more than two adjacent mismatches in the miRNA/target duplex; (3) no adjacent mismatches in positions 2 to 12 of the miRNA/target duplex from the 5′ miRNA end; (4) no mismatches in positions 10 to 11 of the miRNA/target duplex; (5) no more than 2.5 mismatches in positions 1 to 12 of the miRNA/target duplex from the 5′ miRNA end; and (6) the minimum free energy (MFE) of the miRNA/target duplex should be ≥ 75% of the MFE of the miRNA with its perfect complement.

### Differential expression analysis of miRNAs between the leaves and glandular trichomes

To ensure the significance of the difference in miRNA expression, we normalized the expression of miRNAs in the two tissue sources (leaves and glandular trichomes) as transcript *per* million (TPM). Then those miRNAs with a *P*-value<0.05 (adjusted to a corrected *P*-value (q-value) lower than 0.05) and an absolute value of log_2_Ratio>1 were selected as the differentially expressed miRNAs. Target gene prediction of the differentially expressed miRNAs was also conducted to better understand the regulatory roles of the miRNAs. Alignments of the miRNAs to the corresponding target sites are shown in S1 Table.

### GO (Gene Ontology) functional classification and KEGG pathway analysis for the potential targets of the differentially expressed miRNAs

GO is a classification system for gene function, which supplies a set of dynamically updated and controlled vocabulary to comprehensively describe the property of genes and gene products. There are 3 ontologies in GO: molecular function, cellular component and biological process. The basic unit of GO is GO-term, each of which belongs to one type of ontology. Therefore, to classify the function distribution of the potential targets of the differentially expressed miRNAs genes, the Blast2GO program was used to obtain their GO annotations [46] and the WEGO software to obtain their GO functional classifications [47]. The GO enrichment analysis of the targets was conducted and GO terms with a corrected *P*-value ≤ 0.05 were defined as significantly enriched terms. KEGG is a public database regarding metabolic pathways [48]. The target genes were mapped to the KEGG database to identify what pathways in which those targets of the differentially expressed miRNAs are involved.

### Real-time quantitative PCR (RT-qPCR)

Stem-loop RT-qPCR was employed to validate the gene expression data from the Illumina sequencing according to the method previously described by Chen *et al*. [49]. The primers used for this part of the experiment were listed in S2 Table. First-strand cDNA synthesis was performed using RevertAid Reverse Transcriptase (Thermo Scientific). The reaction was carried out at 42°C for 60 min followed by incubation at 70°C for 10 min, and then held at 4°C thereafter. RT-qPCR was conducted using the FastStart Universal SYBR Green Master (Roche) and ABI 7500 Real-Time PCR System according to the manufacturer’s instructions. The reactions were undertaken at 95°C for 10 min for one cycle; at 95°C for 15s, then at 62°C for 1 min for 40 cycles. All reactions were performed in three independent biological samples with three technical repeats. The melting curve was generated to test the specificity of PCR products and avoid the amplicons only from primers themselves. The actin gene of *X*. *strumarium* (GenBank accession no.JF434698) was used as an internal standard to normalize the variation in each sample manipulation and the results were analyzed using the comparative 2^-ΔΔ*Ct*^ method to quantify the relative expression [50].

## Results

### High-throughput sequencing analysis of small RNAs

**Fig 1:**
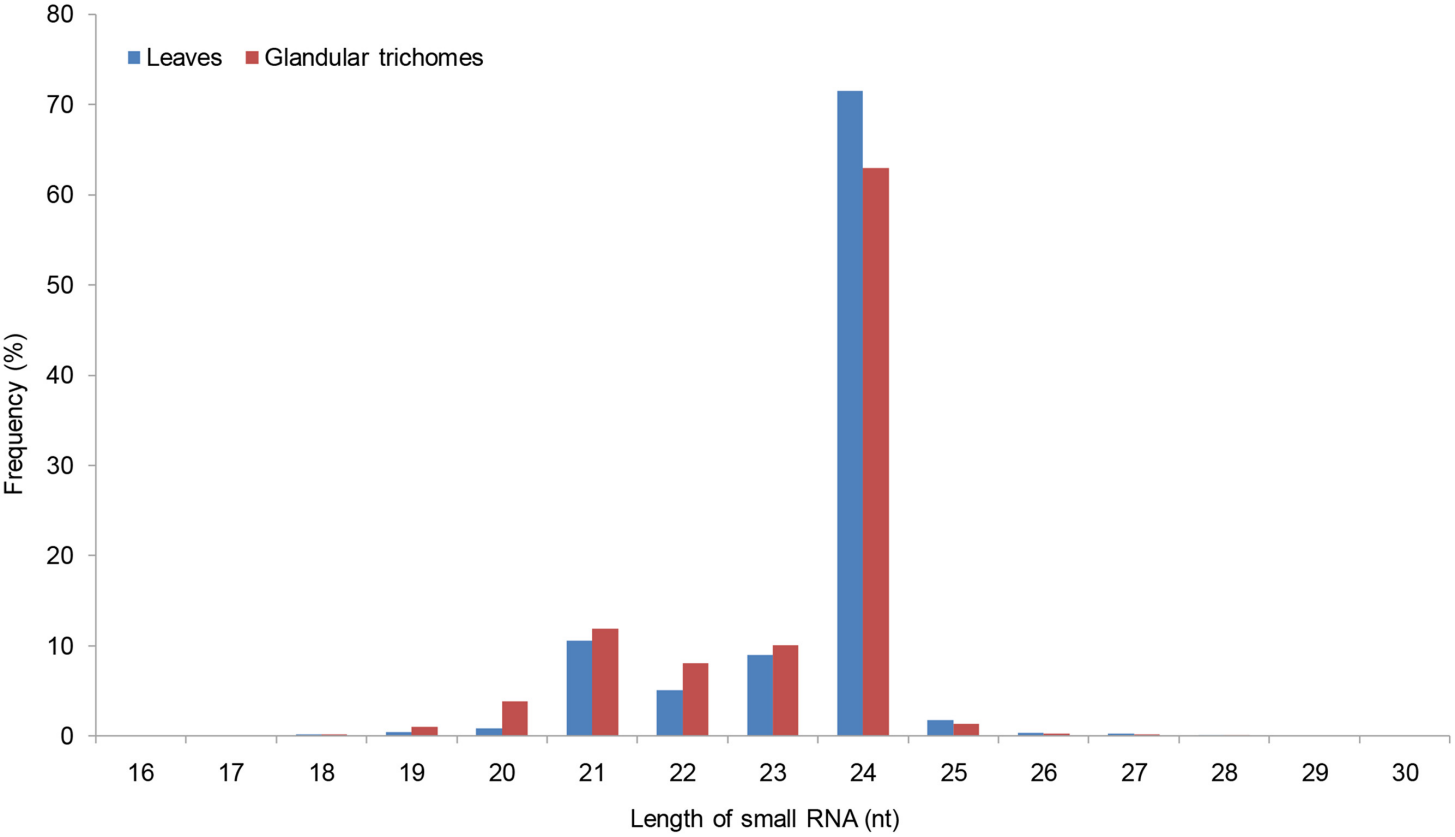
Size distribution of the miRNAs from the leaves and glandular cells.

In total, 12,325,132 raw reads for the leaves and 9,076,601 raw reads for the glandular trichomes were initially generated. After data preprocessing, 12,152,212 and 8,988,274 clean reads for the leaves and glandular trichomes remained for the analysis, generating 7,261,121 and 4,842,894 total unique sequences for the leaves and glandular trichomes, respectively. 6,193,697 and 3,775,470 unique sequences (85.3% and 77.96% of the total unique sequences) were specific to the leaves and glandular trichomes (Table 1). This was indicative of the diversity of small RNA sequences in each tissue source. Little difference was found in the length distribution of the sequences from both tissue sources: the most abundant was the 24 nt small RNAs, accounting for more than 60% of the total reads, followed by 21 nt small RNAs, and small RNAs with a length of 23 nt (Fig 1). In addition, 220,115 (3.03%) and 247,453 (5.11%) unique sequences for the leaves and glandular trichomes matched to the *X*. *strumarium* transcriptome data. After annotating and removing the non-coding RNAs, including rRNAs, tRNAs, snRNAs, and snoRNAs, 37,490 (0.52%) and 33,664 (0.7%) reads for the leaves and glandular trichomes were left for the identification of conserved miRNAs, and 7,138,288 (98.31%) and 4,735,851 (97.79%) unannotated reads for the leaves and glandular trichomes were used to predict novel miRNAs (Table 2).

**Table 1:** Statistics of small RNA sequencing.

Type	Leaves		Glandular trichomes	
	count	%	count	%
total raw reads	12,325,132	—	9,076,601	—
high quality reads	12,195,632	100	9,021,559	100
3'adapter null reads	16,018	0.13	14,806	0.16
insert null reads	881	0.01	638	0.01
5'adapter contaminants	17,105	0.14	9797	0.11
smaller than 18nt reads	7103	0.06	5576	0.06
polyA reads	2313	0.02	2468	0.03
clean reads	12,152,212	99.64	8,988,274	99.63
total unique reads	7,261,121	—	4,842,894	—
tissue_specific unique reads	6,193,697	85.30^a^	3,775,470	77.96^a^

^a^The percentage of the tissue_specific unique reads for the respective tissue source.

**Table 2:** Distribution of small RNAs among different categories in leaves and glandular trichomes of *X*. *strumarium*.

Type	Unique small RNAs		Total small RNAs	
	Leaves	Glandular trichomes	Leaves	Glandular trichomes
total reads	7,261,121(100%)	4,842,894(100%)	12,152,212(100%)	8,988,274(100%)
matched reads^a^	220,115(3.03%)	247,453(5.11%)	1,474,980(12.14%)	1,819,939(20.25%)
miRNA	37,490(0.52%)	33,664(0.70%)	719,520(5.92%)	521,259(5.80%)
rRNA	75,056(1.03%)	60,161(1.24%)	388,404(3.20%)	405,948(4.52%)
snRNA	1003(0.01%)	1239(0.03%)	1493(0.01%)	2317(0.03%)
snoRNA	347(0%)	414(0.01%)	478(0%)	608(0.01%)
tRNA	8937(0.12%)	11,565(0.24%)	38287(0.32%)	339,486(3.78%)
unannotated	7,138,288(98.31%)	4,735,851(97.79%)	11,004,030(90.55%)	7,718,656(85.87%)

^a^The reads that were matched to the *X*. *strumarium* transcriptome.

### Identifying conserved miRNAs in both tissue sources

**Fig 2:**
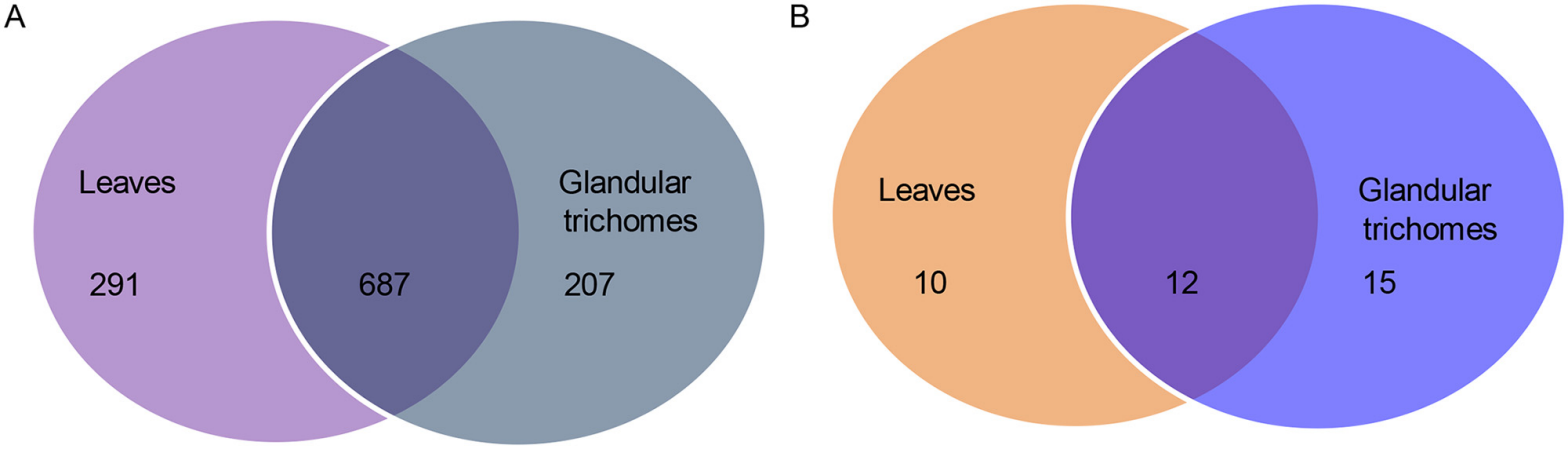
Proportion of identified miRNAs in the leaves and glandular trichomes presented in a Venn diagram. The miRNAs in the diagram consist of three portions: miRNAs that are exclusively present in leaves, miRNAs that are exclusively present in glandular trichomes, and miRNAs present in both tissue sources. (A) conserved miRNAs; (B) novel miRNAs.

In *X*. *strumarium*, no miRNA has been reported at the time of drafting this manuscript. We identified 978 conserved miRNA families with 745,146 total reads in the leaves and 894 miRNA families with a total of 550,246 reads in the glandular trichomes (S3 Table). There were 687 conserved miRNA families expressed in both tissue sources (Fig 2A), of which miR5565 was the most abundant miRNA family with 338,261 reads in the leaves and 249,096 in the glandular trichomes. The expression levels of a few other miRNAs, such as miR166, miR167, miR172, miR398, and miR156 were also very high in both samples, while some miRNAs, including miR2084, miR2670, miR2875 and miR2950, were expressed in extremely low abundance with only less than five reads.

### Identifying potential novel miRNAs in *X*. *strumarium*

Based on the criteria described in the section of Materials and Methods, 22 potential novel miRNAs for the leaves and 27 for the glandular trichomes were identified in both tissue sources with at least five reads (S4 Table). Of these, only 12 novel miRNAs appeared in both samples (Fig 2B), suggesting that the expression profiling of novel miRNAs was different between the leaves and glandular trichomes.

**Fig 3:**
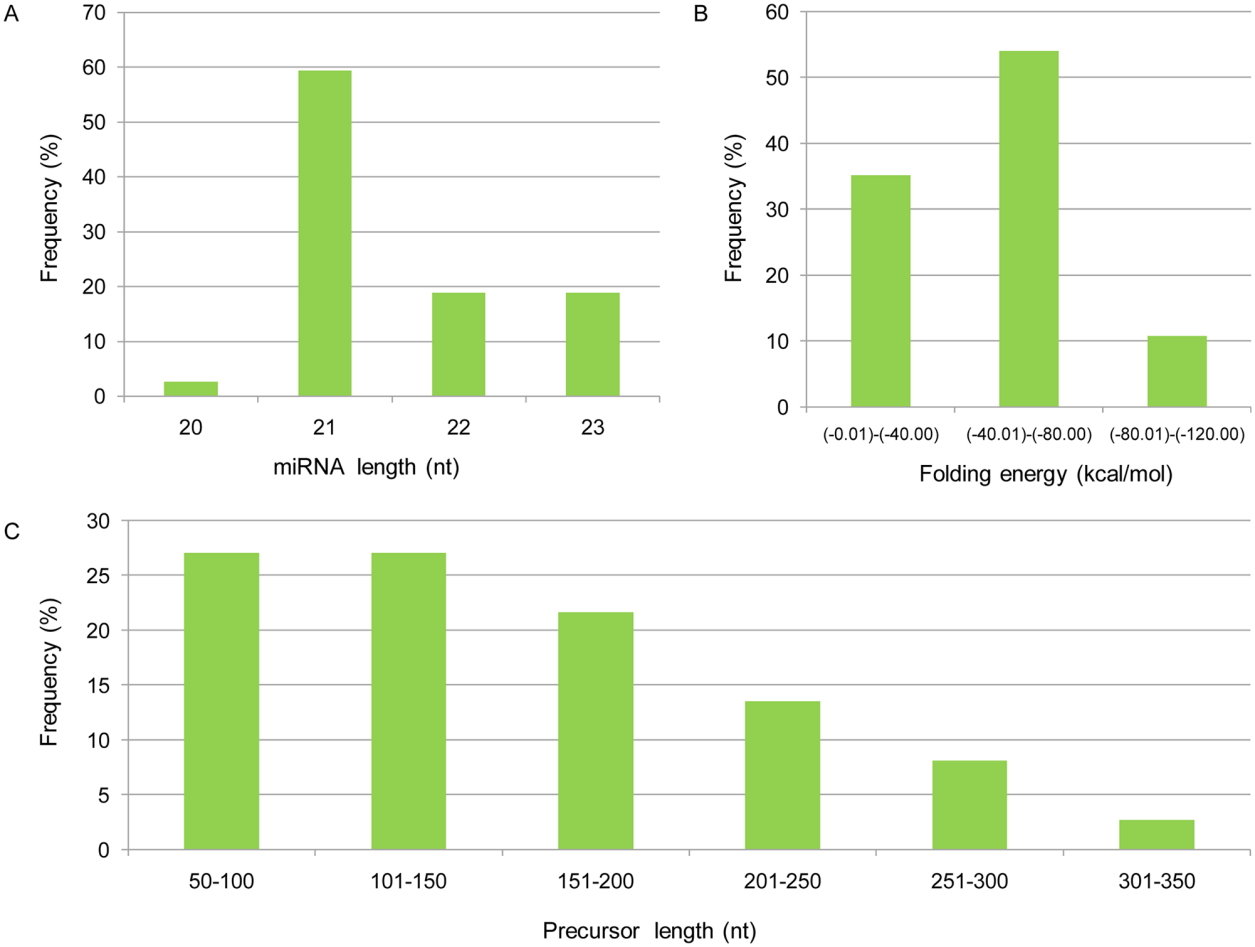
Summary of potential novel miRNAs identified in *X*. *strumarium*. (A) Length frequency for the identified novel miRNAs. (B) Folding energy frequency of precursors for the potential novel miRNAs. (C) Length frequency of precursors for the potential novel miRNAs.

**Fig 4:**
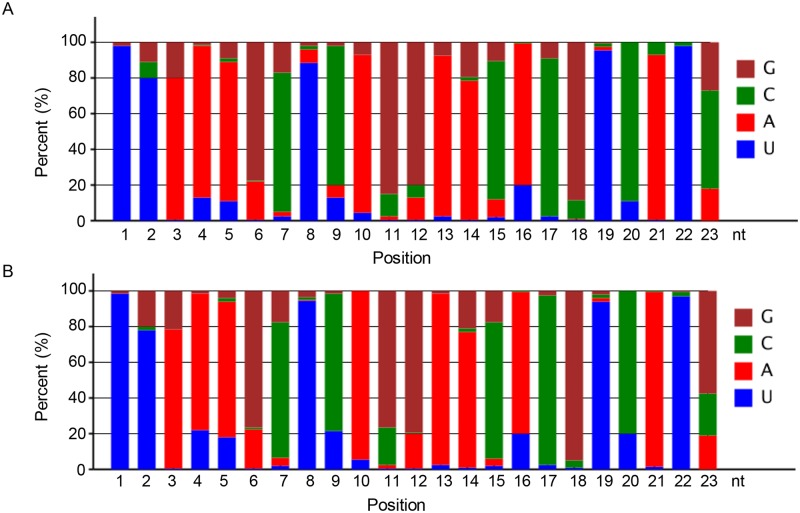
Nucleotide preference at each position of novel miRNAs. (A) miRNA nucleotide bias of novel miRNAs in leaves; (B) miRNA nucleotide bias of novel miRNAs in glandular trichomes.

The identified novel miRNAs ranged from 20 to 23 nt, with 21nt being the most abundant (59.46%) (Fig 3A). The length of the predicted precursors for the novel miRNAs were 66 to 323 nt, with that the majority was between 50 and 150 nt (54.06%) (Fig 3C). The folding energy of these hairpin structures for the precursors of novel miRNAs was -19.7 to -101.8 kcal/mol, which most values within the range of -40 to -80 kcal/mol (54.05%) (Fig 3B). These results were similar to those observed in Chinese cabbage, *Arabidopsis thaliana*, *Oryza sativa* and *Arachis hypogaea* [51–53]. The nucleotide bias analysis showed that novel miRNAs from both tissue sources had the similar tendency on the nucleotide bias at certain key positions, for example, a strong preference for adenosine (A) at the tenth position and for uridine (U) at the first position(Fig 4), which are the typical features of miRNAs[54, 55].

### Target prediction of conserved and novel miRNAs in *X*. *strumarium*

Target genes for the conserved and novel miRNAs were predicted to better understand the biological functions of the identified miRNAs in *X*. *strumarium*. In total, we found 4,071 target genes for 544 conserved miRNAs and 116 target genes for 26 novel miRNAs in *X*. *strumarium*, with an average of 7.48 and 4.46 targets *per* conserved and novel miRNA (S5 Table). To annotate these potential targets, a BlastX search against the NCBI protein database with an *E* value lower than 10^−5^ was performed. Some targets were annotated as transcription factors, including WRKY, Basic helix–loop–helix (bHLH), SQUAMOSA Promoter Binding Protein-Like (SPL) and basic leucine zipper motif (bZIP) proteins. Other target genes included those involved in signal transduction, metabolism, stress response and those with unknown functions. The expression levels of these targets between the leaves and glandular trichomes were also compared (S5 Table).

### The identification of the miRNAs differentially expressed between the two tissue sources

**Fig 5:**
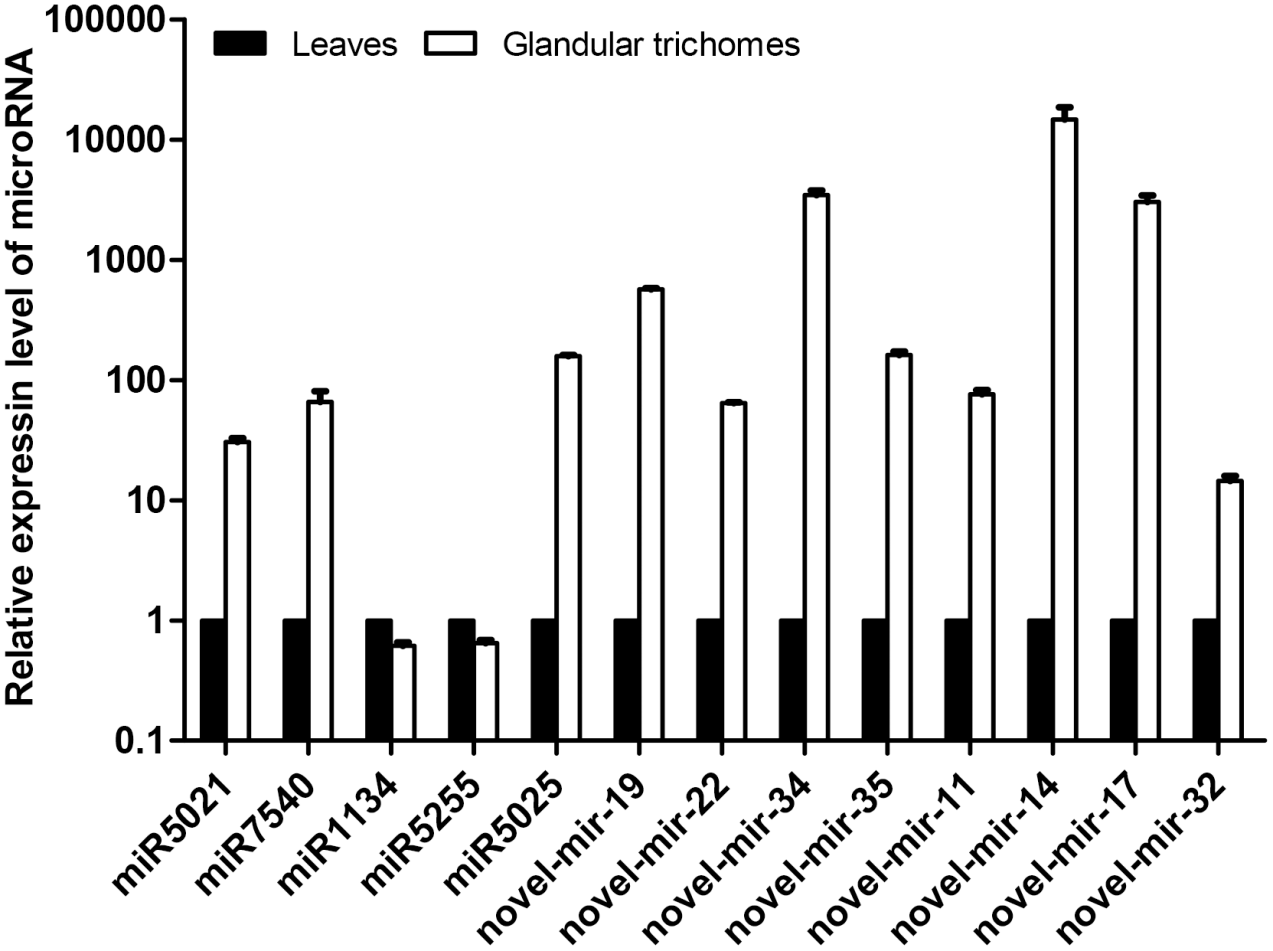
RT-qPCR data for the transcript abundance of some miRNAs in the leaves and glandular trichomes. The miRNA levels were normalized to an internal control (actin) and expressed relative to the values of leaves (control), which were given an arbitrary value of 1. Error bars indicate the standard deviation of three biological replicates.

The total of 512 miRNAs, including 494 conserved and 18 novel miRNAs, were found to be differentially expressed between the two tissue sources. Among them, 262 conserved and 13 novel miRNAs were up-regulated, and 232 conserved and five novel miRNAs were down-regulated in the glandular trichomes (S6 Table). To validate the miRNA expression data from the sequencing, the expression levels of 13 differentially expressed miRNAs, including eight novel miRNAs and five conserved miRNAs, were measured using RT-qPCRs. As was shown in Fig 5, the expression trend of most of the miRNAs, except for miR1134, was consistent with the Illumina sequencing results, meaning that the gene expression data of miRNAs by the sequencing technique was credible.

### Target prediction of the differentially expressed miRNAs

Based on the *X*. *strumarium* transcriptome, a total of 3,307gene targets were identified for those differentially expressed miRNAs (S6 Table). Among these targets, some encode transcription factors such as v-myb avian myeloblastosis viral oncogene homolog (MYB), WRKY, bHLH, APETALA2/ethylene-responsive factor (AP2/ERF), bZIP and SPL proteins. For instance, the unique genes, including CL6103.Contig1 targeted by miR5072, CL1989.Contig2_All targeted by miR7539 and Unigene12046_All targeted by miR1850, displayed high similarities to WRKY proteins. WRKY transcription factors have been reported to play roles in regulating the biosynthesis of terpenoids [56–58]. By mapping those targets to the KEGG pathway database, we were able to find that some targets seemed to encode putative enzymes in terpenoid biosynthesisin *X*. *strumarium* (the ones highlighted by yellow color in S7 Table), especially in sesquiterpene biosynthesis (Table 3). For example, the upstream enzymes in the pathways of terpenoid biosynthesis, including 1-deoxy-D-xylulose 5-phosphate synthase (DXS), 3-hydroxy-3-methylglutaryl coenzyme A reductase (HMGR), isopentenyl diphosphate (IPP)/dimethylallyl diphosphate (DMAPP) synthase (IDS), and isopenteyl diphosphate isomerase (IDI), were predicted to be targeted by miR7539, miR5021 and miR1134. DXS, HMGR, IDS, and IDI are the enzymes involved in the biosynthesis of IPP and DMAPP, the common precursors of all the terpenoids [59]. In particular, HMGR is a rate-limiting enzyme of the pathway to synthesize IPP and DMAPP [60]. The target by miR6435 is homologous to germacrene A oxidase (GAO), the first key enzyme in the pathway to the biosynthesis of xanthanolides [61]. Interestingly, xanthanolides have been considered to be the major active compounds in *X*. *strumarium* [62]. In addition, some targets are homologs to the enzymes in the biosynthesis of di-, or tri-terpenoids. For example, the beta-amyrin synthase targed by miR5491 and ent-kaurene synthase targeted by miR6449 are key enzymes that catalyze the formation of the most common triterpene *β*-Amyrin and diterpene ent-kaurene [63, 64].

**Table 3:** Target genes for differentially expressed miRNAs involved in terpenoids biosynthesis.

microRNAs	Target gene candidates	Annotation	Biosynthetic pathway
miR6435	Unigene22477_All	Germacrene A oxidase	sesquiterpenoid[61]
miR5255	Unigene26141_All	Squalene epoxidase	triterpenoid[65, 66]
miR5255	Unigene26143_All	Squalene epoxidase	triterpenoid
miR5255	Unigene26144_All	Squalene epoxidase	triterpenoid
miR5255	Unigene26145_All	Squalene epoxidase	triterpenoid
miR5255	Unigene26146_All	Squalene epoxidase	triterpenoid
miR5491	CL1191.Contig1_All	beta-amyrin synthase	triterpenoid[64]
miR5491	CL1191.Contig2_All	beta-amyrin synthase	triterpenoid
miR5491	CL1191.Contig3_All	beta-amyrin synthase	triterpenoid
miR5491	CL1191.Contig5_All	beta-amyrin synthase	triterpenoid
miR5491	Unigene18850_All	beta-amyrin synthase	triterpenoid
miR5021	CL12255.Contig3_All	HMGR	terpenoid backbone [59]
miR1134	CL12255.Contig3_Al	HMGR	terpenoid backbone
miR5021	CL3919.Contig4_All	IDS	terpenoid backbone [59]
miR5021	Unigene24678_All	IDI	terpenoid backbone [59]
miR5021	Unigene23634_All	IDI	terpenoid backbone
miR7539	CL4414.Contig1_All	DXS	terpenoid backbone [59]
miR7540	CL2999.Contig1_All	R-linalool synthase	monoterpenoid[67]
miR5183	CL5257.Contig2_All	gibberellin 3-oxidase	diterpenoid[68]
miR6449	CL5429.Contig1_All	ent-kaurene synthase	diterpenoid[63]
miR6449	CL5429.Contig7_All	ent-kaurene synthase	diterpenoid
miR6449	CL5429.Contig8_All	ent-kaurene synthase	diterpenoid

**Fig 6:**
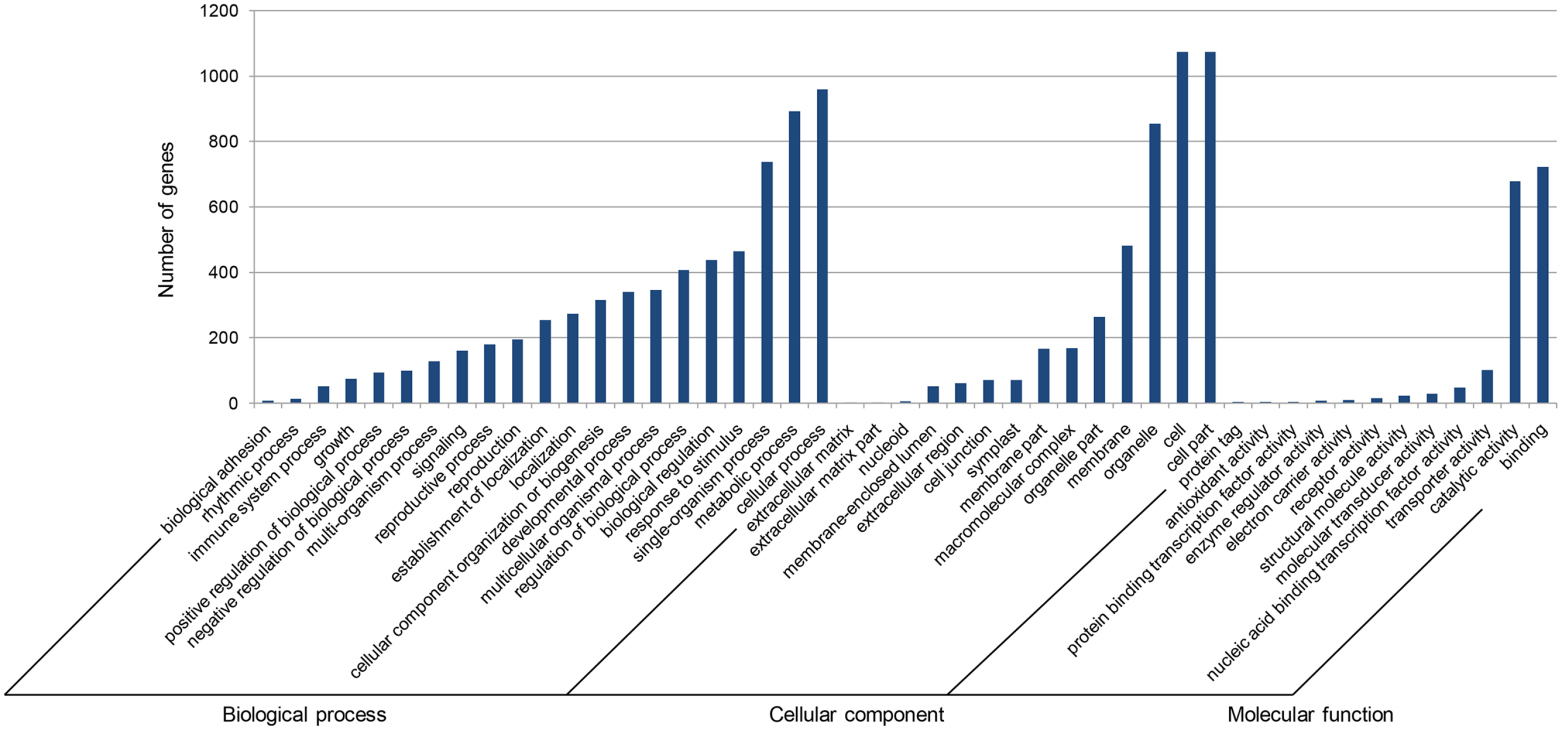
GO functional classification for the predicted targets by the differentially expressed miRNAs. X-axis, the three main GO categories and 47 GO terms assigned for the differentially expressed miRNA targets; Y-axis, the gene numbers corresponding to the GO terms.

GO analysis showed that these targets could be summarized into three main categories and classified into 47 functional groups (Fig 6). Based on the biological process category, the majority of the targets were involved in “cellular process”, “metabolic process” and “single-organism process”. In the case of molecular functions, a large number of genes were grouped into “binding” and “catalytic activity”. While in the cellular component, the genes were mostly related to “cell”, “cell part” and “organelle”. The GO enrichment analysis showed that the terms “mitochondrial respiratory chain complex IV” and “respiratory chain complex IV” are overrepresented in the cellular component. For the molecular function, the majority of genes were found to be involved in the “oxidoreductase activity” and “cytochrome-c oxidase activity”. The GO terms “heme a metabolic process” and “heme a biosynthetic process” account for a large proportion in the biological process, which indicated that the miRNAs might play roles in modulating plant metabolic processes (S8 Table).

## Discussion

Plant miRNAs have been reported to be involved in a variety of important processes, including development, signal transduction, and responses to environmental stresses [12]. There was also evidence to show that miRNAs function in regulating secondary metabolic activities. For example, the molecule miRNA156 is involved in the regulation of flavonoid biosynthesis in *Arabidopsis thaliana* [21]. Glandular trichomes are one type of specialized structure in synthesizing a wide range of plant secondary metabolites [69], in *X*. *strumarium*, they are also the primary sites for accumulating xanthanolides, the compounds with multiple bioactivities [40]. We hypothesized that miRNA expression in glandular cells might play roles in regulating the biosynthesis of secondary metabolites in *X*. *strumarium* such as xanthanolides. However, to the best of our knowledge, no any information is available for the miRNAs from glandular trichomes of any plant species. As the beginning to address this hypothesis, glandular trichomes were physically isolated from the young leaves of *X*. *strumarium* in this study and large sets of miRNAs in this particular structure were identified using a high-throughput sequencing technology. A database for miRNAs from its intact young leaves was also constructed and used as a comparison. A total of 894 conserved miRNAs and 27 novel miRNAs were successfully identified from the glandular trichomes. The expression levels of more than 50% of these miRNAs seem to be up- or down-regulated in the glandular trichomes compared to intact leaves. The reliability of the gene expression data was confirmed by the Q-RT-PCR analysis of the five conserved and eight novel miRNAs that were randomly selected. The expression of, novel-mir-14, novel-mir-17, and novel-mir-34 is very high in glandular trichomes and more than 1000 folds to those in intact leaves, indicating that they may have physiological functions in this specialized structure. With respects to the miRNAs with the highest abundance in the glandular cells, they may be glandular trichome-specifically expressed or transported into the trichomes from the other parts of the leaves.

To understand the regulatory roles of miRNAs, it is essential to predict and annotate its target mRNAs. Based on the feature that plant miRNAs are perfectly complementary to their targets, miRNA target genes can be predicted by a bioinformatics approach [3, 7]. Using the bioinformatics tool, we were able to identify that the targets by the glandular trichomes conserved miRNAs included transcription factors and non-transcriptional factor proteins (S5 Table). Several transcription factors were predicted to be targeted by the same conserved miRNA molecule, for example, miR7539 targets MYB, bHLH, WAKY, zinc finger, DNA Binding With One Finger(DOF), SPL, and bZIP transcription factors and miR5658 targets MYB, bHLH, zinc finger, and bZIP transcription factors, suggesting that these miRNAs may play multiple roles in diverse physiological processes. Non-transcriptional factor proteins, such as DXS, HMGR, IDS and IDI, were predicted to be targets of miR7539, miR5021 and miR1134, respectively. These targets are essential enzymes in upstream isoprenoid pathway to produce IPP and DMAPP, the common precursors for all the downstream end terpenoids. In particular, HMGR is a key regulatory enzyme that controls the amounts of isoprenoids [60]. These data suggested that miR7539, miR5021 and miR1134 might be involved in regulating terpenoid biosynthesis by targeting upstream terpenoid pathway genes. Some miRNAs target putative downstream enzymes in the biosynthesis of mono-, sesqui-, di-, and tri-terpenoids. They were R-linalool synthase, gibberellin 3-oxidase, ent-kaurene synthase, squalene epoxidase, beta-amyrin synthase, and germacreneA oxidase, which were targeted by miR7540, miR5183, miR6449, miR5255, miR5491, and miR6435, respectively (Table 3). Most interestingly, germacrene A oxidase (GAO) targeted by miR6435 is a key P450 involved in the biosynthesis of xanthanolides[61], which was previously reported to be active molecules to contribute to the pharmacological property of *X*. *strumarium* [30, 34, 70]. Gene expression data from the miRNA-sequencing showed that the molecule miR6435 is glandular trichome-specifically expressed (S6 Table), which is also consistent with the feature that glandular trichomes are the primary sites to synthesize xanthanolides. The data allowed us to hypothesize that miR6435 might play a role in the regulation of xanthanolide biosynthesis in *X*. *strumarium* glandular trichomes. Identification of miRNAs which can perfectly bind to their mRNA targets may also provide alternate approach to isolate pathway genes, especially for those pathways that are not elucidated well. The discovery of *X*. *strumarium* glandular trichome miRNAs of this study may help to identify xanthanolide biosynthesis genes. The biosynthetic pathway of xanthanolides is not elucidated yet, especially for its downstream pathway in which cytochrome P450 enzymes are presumably involved. Here, we have tried to identify miRNAs whose targets were P450 mRNAs. In addition to GAO targeted by miR6435, we also have found that the targets of other miRNAs, including miR1512, miR3447, miR5678, miR6283, and miR7539, encode P450s, in particular, of these P450 mRNA sequences, the target mRNA by miR6283 seemed to be trichome-specifically expressed (S5 Table). Thus, it will be of interest to further experimentally perform functional analysis of miR6283 and its target. In contrast to the conserved miRNA targets, none of the targets by novel miRNAs presented in this research were transcription factors and many of them encoded cytochrome c oxidase, ABC transporter, and protein kinases, suggesting their roles in oxidation-reduction processes, transport, and signal transduction.

## Conclusions

In conclusion, this is the first comprehensive identification of miRNAs from the plant glandular trichomes, the specialized structure to synthesize a wide range of medicinal molecules. We have been able to identify miRNAs and their mRNA targets that are trichome-specifically expressed. The data of this study provide the starting point to further investigation to elucidate the miRNAs regulatory mechanism underlying the biosynthesis of secondary metabolites, especially terpenoids, in *X*. *strumarium* glandular cells.

## Supporting Information

S1 Table Alignments of the miRNAs to the target sites. (XLSX)

S2 Table Primers used in this study. (XLSX)

S3 Table Analysis of identified conserved miRNA families in leaves and glandular trichomes of *X*. *strumarium*. (XLSX)

S4 Table Analysis of novel miRNAs in leaves and glandular trichomes of *X*. *strumarium*. (XLSX)

S5 Table Target prediction of the *X*. *strumarium* miRNAs and expression analysis of the targets between the leaves and glandular trichomes of the plant. (XLSX)

S6 Table Target prediction of conserved and novel miRNAs differentially expressed in leaves and glandular trichomes. (XLSX)

S7 Table KEGG pathway analysis of genes for differentially expressed miRNAs. (XLSX)

S8 Table GO enrichment analysis of the targets by the differentially expressedmiRNAs. (XLSX)

## References

[1] Bartel DP. MicroRNAs: genomics, biogenesis, mechanism, and function. Cell. 2004;116(2):281–97. 14744438 10.1016/s0092-8674(04)00045-5

[2] Ha M, Kim VN. Regulation of microRNA biogenesis.Nat Rev Mol Cell Biol. 2014;15(8):509–24. 10.1038/nrm3838 25027649

[3] Allen E, Xie Z, Gustafson AM, Carrington JC. microRNA-directed phasing during trans-acting siRNA biogenesis in plants. Cell. 2005;121(2):207–21. 10.1016/j.cell.2005.04.004 15851028

[4] Aukerman MJ, Sakai H. Regulation of flowering time and floral organ identity by a MicroRNA and its *APETALA2*-like target genes. Plant Cell. 2003;15(11):2730–41. 10.1105/tpc.016238 14555699 PMC280575

[5] Bartel DP. MicroRNAs: Target Recognition and Regulatory Functions. Cell. 2009;136(2):215–33. 19167326 10.1016/j.cell.2009.01.002 PMC3794896

[6] Chen X. A microRNA as a translational repressor of *APETALA2* in *Arabidopsis* flower development. Science. 2004;303(5666):2022–5. 12893888 10.1126/science.1088060 PMC5127708

[7] Schwab R, Palatnik JF, Riester M, Schommer C, Schmid M, Weigel D. Specific effects of microRNAs on the plant transcriptome. Dev Cell. 2005;8(4):517–27. 15809034 10.1016/j.devcel.2005.01.018

[8] Frazier T, Zhang B. Identification of Plant microRNAs Using Expressed Sequence Tag Analysis In: Pereira A, editor. Plant Reverse Genetics. Methods in Molecular Biology. New York: Humana Press; 2011 pp. 13–25. 10.1007/978-1-60761-682-5_2 20931369

[9] Jagadeeswaran G, Saini A, Sunkar R. Biotic and abiotic stress down-regulate miR398 expression in *Arabidopsis* . Planta. 2009;229(4):1009–14. 10.1007/s00425-009-0889-3 19148671

[10] Phillips JR, Dalmay T, Bartels D. The role of small RNAs in abiotic stress. FEBS Lett. 2007;581(19):3592–7. 10.1016/j.febslet.2007.04.007 17451688

[11] Yang T, Xue L, An L. Functional diversity of miRNA in plants. Plant Sci. 2007;172(3):423–32. 10.1016/j.plantsci.2006.10.009

[12] Zhang B, Pan X, Cobb GP, Anderson TA. Plant microRNA: a small regulatory molecule with big impact. Dev Biol. 2006;289(1):3–16. 10.1016/j.ydbio.2005.10.036 16325172

[13] Robert-Seilaniantz A, Bari R, Jones JD. A biotic or abiotic stress? In:Pareek A, Sopory SK, Bohnert HJ, Govindjee, editors. Abiotic Stress Adaptation in Plants. Dordrecht: Springer; 2010pp. 103–122.

[14] Schommer C, Palatnik JF, Aggarwal P, Chételat A, Cubas P, Farmer EE, et al Control of jasmonate biosynthesis and senescence by miR319 targets. PLoS Biol. 2008;6(9):e230 10.1371/journal.pbio.0060230 18816164 PMC2553836

[15] Wu B, Wang M, Ma Y, Yuan L, Lu S. High-throughput sequencing and characterization of the small RNA transcriptome reveal features of novel and conserved microRNAs in Panax ginseng. PLoS One. 2012;7(9):e44385 22962612 10.1371/journal.pone.0044385 PMC3433442

[16] Boke H, Ozhuner E, Turktas M, Parmaksiz I, Ozcan S, Unver T. Regulation of the alkaloid biosynthesis by miRNA in opium poppy. PlantBiotechnol J. 2015;13(3):409–20. 10.1111/pbi.12346 25735537

[17] Hao DC, Yang L, Xiao PG, Liu M. Identification of Taxus microRNAs and their targets with high‐throughput sequencing and degradome analysis. Physiol Plant. 2012;146(4):388–403. 10.1111/j.1399-3054.2012.01668.x22708792 22708792

[18] Prakash P, Ghosliya D, Gupta V. Identification of conserved and novel microRNAs in Catharanthusroseus by deep sequencing and computational prediction of their potential targets. Gene. 2015;554(2):181–95. 10.1016/j.gene.2014.10.046 25445288

[19] Robert-Seilaniantz A, MacLean D, Jikumaru Y, Hill L, Yamaguchi S, Kamiya Y, et al The microRNA miR393 re-directs secondary metabolite biosynthesis away from camalexin and towards glucosinolates. Plant J. 2011;67(2):218–31. 10.1111/j.1365-313X.2011.04591.x 21457368

[20] Ng DW, Zhang C, Miller M, Palmer G, Whiteley M, Tholl D, et al cis-and trans-Regulation of miR163 and target genes confers natural variation of secondary metabolites in two Arabidopsis species and their allopolyploids. Plant Cell. 2011;23(5):1729–40. 10.1105/tpc.111.083915 21602291 PMC3123960

[21] Gou J-Y, Felippes FF, Liu C-J, Weigel D, Wang J-W. Negative regulation of anthocyanin biosynthesis in Arabidopsis by a miR156-targeted SPL transcription factor.Plant Cell. 2011;23(4):1512–22. 10.1105/tpc.111.084525 21487097 PMC3101539

[22] Hammerschmidt D. Xanthium strumarium. J Lab Clin Med. 1998;132(1):86 9665377 10.1016/s0022-2143(98)90030-0

[23] Chandel S, Bagai U, Vashishat N. Antiplasmodial activity of *Xanthium strumarium* against Plasmodium berghei-infected BALB/c mice. Parasitol Res. 2012;110(3):1179–83. 10.1007/s00436-011-2611-1 21847597

[24] Gautam R, Saklani A, Jachak SM. Indian medicinal plants as a source of antimycobacterial agents. J Ethnopharmacol. 2007;110(2):200–34. 10.1016/j.jep.2006.12.031 17276637

[25] Ma Y-T, Huang M-C, Hsu F-L, Chang H-F. Thiazinedione from *Xanthium strumarium* .Phytochemistry. 1998;48(6):1083–5. 10.1016/S0031-9422(98)00084-3 16556487

[26] Yin MH, Kang DG, Choi DH, Kwon TO, Lee HS. Screening of vasorelaxant activity of some medicinal plants used in Oriental medicines. J Ethnopharmacol. 2005;99(1):113–7. 10.1016/j.jep.2005.02.013 15848029

[27] Abdei-Mogib M, Dawidar A, Metwally M, Abou-Elzahab M. Xanthanolides from *Xanthium spinosum* . Phytochemistry. 1991;30(10):3461–2. 10.1016/0031-9422(91)83230-I

[28] Ahmed AA, Jakupovic J, Bohlmann F, Regaila H, Ahmed A. Sesquiterpene lactones from *Xanthium pungens* . Phytochemistry. 1990;29(7):2211–5. 10.1016/0031-9422(90)83040-8

[29] Mahmoud AA. Xanthanolides and Xanthane Epoxide Derivatives from *Xanthium strumarium* . Planta Med. 1998;64(08):724–7. 17253317 10.1055/s-2006-957566

[30] Rodriguez E, Towers G, Mitchell J. Biological activities of sesquiterpene lactones. Phytochemistry. 1976;15(11):1573–80. 10.1016/S0031-9422(00)97430-2

[31] Saxena V, Mondal S. A xanthanolide from *Xanthium strumarium* .Phytochemistry. 1994;35(4):1080–2. 10.1016/S0031-9422(00)90678-2

[32] Harada A, Sakata K, Ina H, Ina K. Isolation and Identification of Xanthatin as an Anti-Attaching Repellent against Blue Mussel. Agric Biol Chem. 1985;49(6):1887–8. WOS:A1985AKZ5200054

[33] Kawazu K, Nakajima S, Ariwa M. Xanthumin and 8-epi-xanthatin as insect development inhibitors from *Xanthium canadense* Mill. Experientia. 1979;35(10):1294–5. 115705 10.1007/BF01963966

[34] Kim H, Lee I, Yeo S, Seong L, Yu T. Isolation and Characterization of Antitumor Agents from *Xanthium strumarium* L. Korean J Biotechnol Bioeng. 2003;18:324–8.

[35] Lavault M, Landreau A, Larcher G, Bouchara J-P, Pagniez F, Le Pape P, et al Antileishmanial and antifungal activities of xanthanolides isolated from *Xanthium macrocarpum* . Fitoterapia. 2005;76(3):363–6. 10.1016/j.fitote.2005.03.019 15890467

[36] Roussakis C, Chinou I, Vayas C, Harvala C, Verbist J. Cytotoxic activity of xanthatin and the crude extracts of *Xanthium strumarium* . Planta Med. 1994;60(5):473 7997481 10.1055/s-2006-959537

[37] Sato Y, Oketani H, Yamada T, Singyouchi KI, Ohtsubo T, Kihara M, et al A Xanthanolide with Potent Antibacterial Activity against Methicillin-resistant *Staphylococcus aureus* .J Pharm Pharmacol. 1997;49(10):1042–4. 10.1111/j.2042-7158.1997.tb06038.x 9364417

[38] Nibret E, Youns M, Krauth-Siegel RL, Wink M. Biological activities of xanthatin from *Xanthium strumarium* leaves. Phytother Res. 2011;25(12):1883–90. 10.1002/ptr.3651 21953905

[39] Ramírez-Erosa I, Huang Y, Hickie RA, Sutherland RG, Barl B. Xanthatin and xanthinosin from the burs of *Xanthium strumarium* L. as potential anticancer agents. Can J PhysiolPharmacol. 2007;85(11):1160–72. . 18066118 10.1139/Y07-104

[40] Chen F, Hao F, Li C, Gou J, Lu D, Gong F, et al Identifying three ecological chemotypes of *Xanthium strumarium* glandular trichomes using a combined NMR and LC-MS method. PLoS One. 2013;8(10):e76621 10.1371/journal.pone.0076621 24098541 PMC3788720

[41] Li R, Yu C, Li Y, Lam T-W, Yiu S-M, Kristiansen K, et al SOAP2: an improved ultrafast tool for short read alignment. Bioinformatics. 2009;25(15):1966–7. 10.1093/bioinformatics/btp336 19497933

[42] Benson DA, Cavanaugh M, Clark K, Karsch-Mizrachi I, Lipman DJ, Ostell J, et al GenBank. Nucleic Acids Res. 2013;41(D1):D36–D42. 10.1093/nar/gks1195 27899564 PMC5210553

[43] Griffiths-Jones S, Bateman A, Marshall M, Khanna A, Eddy SR. Rfam: an RNA family database. Nucleic Acids Res. 2003;31(1):439–41. 10.1093/nar/gkg006 12520045 PMC165453

[44] Griffiths-Jones S, Grocock RJ, Van Dongen S, Bateman A, Enright AJ. miRBase: microRNA sequences, targets and gene nomenclature. Nucleic Acids Res. 2006;34(suppl1):D140–D4. 10.1093/nar/gkj112 16381832 PMC1347474

[45] Chen M, Zhang X, Liu J, Storey KB. High-throughput sequencing reveals differential expression of miRNAs in intestine from sea cucumber during aestivation. PLoS One. 2013;8(10): e76120 10.1371/journal.pone.0076120 24143179 PMC3797095

[46] Conesa A, Götz S, García-Gómez JM, Terol J, Talón M, Robles M. Blast2GO: a universal tool for annotation, visualization and analysis in functional genomics research. Bioinformatics. 2005;21(18):3674–6. 10.1093/bioinformatics/bti610 16081474

[47] Ye J, Fang L, Zheng H, Zhang Y, Chen J, Zhang Z, et al WEGO: a web tool for plotting GO annotations. Nucleic Acids Res. 2006;34(suppl2):W293–W7. 10.1093/nar/gkl031 16845012 PMC1538768

[48] Kanehisa M, Araki M, Goto S, Hattori M, Hirakawa M, Itoh M, et al KEGG for linking genomes to life and the environment. Nucleic Acids Res. 2008;36(suppl 1):D480–D4. 10.1093/nar/gkm882 18077471 PMC2238879

[49] Chen C, Ridzon DA, Broomer AJ, Zhou Z, Lee DH, Nguyen JT, et al Real-time quantification of microRNAs by stem–loop RT–PCR. Nucleic Acids Res. 2005;33(20):e179–e. 10.1093/nar/gni178 16314309 PMC1292995

[50] Livak KJ, Schmittgen TD. Analysis of relative gene expression data using real-time quantitative PCR and the 2(-Delta Delta C(T)) Method. Methods. 2001;25(4):402–8. 10.1006/meth.2001.1262 11846609

[51] Chi X, Yang Q, Chen X, Wang J, Pan L, Chen M, et al Identification and characterization of microRNAs from peanut (*Arachishypogaea* L.) by high-throughput sequencing. PLoS One. 2011;6(11):e27530 10.1371/journal.pone.00275322110666 22110666 PMC3217988

[52] Wang F, Li H, Zhang Y, Li J, Li L, Liu L, et al MicroRNA expression analysis of rosette and folding leaves in Chinese cabbage using high-throughput Solexa sequencing. Gene. 2013;532(2):222–9. 10.1016/j.gene.2013.09.039 24055726

[53] Zhang B, Pan X, Cannon CH, Cobb GP, Anderson TA. Conservation and divergence of plant microRNA genes. Plant J. 2006;46(2):243–59. 10.1111/j.1365-313X.2006.02697.x 16623887

[54] Mi S, Cai T, Hu Y, Chen Y, Hodges E, Ni F, et al Sorting of small RNAs into Arabidopsis argonaute complexes is directed by the 5’ terminal nucleotide. Cell. 2008;133(1):116–27. 10.1016/j.cell.2008.02.034 18342361 PMC2981139

[55] Jones-Rhoades MW, Bartel DP. Computational identification of plant microRNAs and their targets, including a stress-induced miRNA.Mol Cell. 2004;14(6):787–99. 10.1016/j.molcel.2004.05.027 15200956

[56] Eulgem T, Rushton PJ, Robatzek S, Somssich IE. The WRKY superfamily of plant transcription factors. Trends Plant Sci. 2000;5(5):199–206. 10.1016/S1360-1385(00)01600-9 10785665

[57] Ma D, Pu G, Lei C, Ma L, Wang H, Guo Y, et al Isolation and characterization of AaWRKY1, an *Artemisia annua* transcription factor that regulates the amorpha-4, 11-diene synthase gene, a key gene of artemisinin biosynthesis. Plant Cell Physiol. 2009;50(12):2146–61. 10.1093/pcp/pcp149 19880398

[58] Yang CQ, Fang X, Wu XM, Mao YB, Wang LJ, Chen XY. Transcriptional regulation of plant secondary metabolism. J Integr Plant Biol. 2012;54(10):703–12. 10.1111/j.1744-7909.2012.01161.x 22947222

[59] Ma Y, Yuan L, Wu B, Li X, Chen S, Lu S. Genome-wide identification and characterization of novel genes involved in terpenoid biosynthesis in *Salvia miltiorrhiza* . J Exp Bot. 2012;63(7):2809–23. 10.1093/jxb/err466 22291132 PMC3346237

[60] Choi D, Ward BL, Bostock RM. Differential induction and suppression of potato 3-hydroxy-3-methylglutaryl coenzyme A reductase genes in response to Phytophthorainfestans and to its elicitor arachidonic acid. Plant Cell. 1992;4(10):1333–44. 10.1105/tpc.4.10.1333 . 1283354 PMC160219

[61] Eljounaidi K, Cankar K, Comino C, Moglia A, Hehn A, Bourgaud F, et al Cytochrome P450s from *Cynara cardunculus* L. CYP71AV9 and CYP71BL5, catalyze distinct hydroxylations in the sesquiterpene lactone biosynthetic pathway. Plant Sci. 2014;223:59–68. 10.1016/j.plantsci.2014.03.007 24767116

[62] Kamboj A, Saluja AK. Phytopharmacological review of *Xanthium strumarium* L.(Cocklebur). Int J Green Pharm. 2010;4(3):129.

[63] Hayashi K-i, Kawaide H, Notomi M, Sakigi Y, Matsuo A, Nozaki H. Identification and functional analysis of bifunctional*ent*-kaurene synthase from the moss *Physcomitrella patens* . FEBS Lett. 2006;580(26):6175–81. 10.1016/j.febslet.2006.10.018 17064690

[64] Kushiro T, Shibuya M, Ebizuka Y. β-Amyrin synthase. Eur J Biochem. 1998;256(1):238–44. 9746369 10.1046/j.1432-1327.1998.2560238.x

[65] Jung JD, Park HW, Hahn Y, Hur CG, In DS, Chung HJ, et al Discovery of genes for ginsenoside biosynthesis by analysis of ginseng expressed sequence tags. Plant Cell Rep. 2003;22(3):224–30. 10.1007/s00299-003-0678-6 12920566

[66] Suzuki H, Achnine L, Xu R, Matsuda SP, Dixon RA. A genomics approach to the early stages of triterpene saponin biosynthesis in *Medicago truncatula* . Plant J. 2002;32(6):1033–48. 10.1046/j.1365-313X.2002.01497.x 12492844

[67] Landmann C, Fink B, Festner M, Dregus M, Engel K-H, Schwab W. Cloning and functional characterization of three terpene synthases from lavender (*Lavandulaangustifolia*). Arch BiochemBiophys. 2007; 465(2):417–29. 10.1016/j.abb.2007.06.011 17662687

[68] Chen Y, Hou M, Liu L, Wu S, Shen Y, Ishiyama K, et al The maize DWARF1 encodes a gibberellin 3-oxidase and is dual localized to the nucleus and cytosol. Plant Physiol. 2014;166(4):2028–39. 10.1104/pp.114.247486 25341533 PMC4256885

[69] Duke SO. Glandular trichomes-a focal point of chemical and structural interactions.Int JPlantSci. 1994;155(6):617–20.

[70] Kupchan SM, Eakin M, Thomas A. Tumor inhibitors. 69. Structure-cytotoxicity relations among the sesquiterpene lactones. J Med Chem. 1971; 14(12):1147–52. 10.1021/jm00294a001 5116225

